# Effects of Toxic Leadership Behavior and Emotional Exhaustion on Patient Safety Incident Reporting

**DOI:** 10.1155/jonm/5547937

**Published:** 2026-04-17

**Authors:** Collins Atta Poku, Yvonne Akpene Ahiadzese, Hadassah Korkor Oyortey, Sarah Naa Odarkai Lamptey, Jeremiah Asante, Daisy Afrah Lumor, Doris Hagan

**Affiliations:** ^1^ Department of Nursing, Kwame Nkrumah University of Science and Technology, Kumasi, Ghana, knust.edu.gh; ^2^ Department of Midwifery, Kwame Nkrumah University of Science and Technology, Kumasi, Ghana, knust.edu.gh; ^3^ Department of Public Health Nursing, Kwame Nkrumah University of Science and Technology, Kumasi, Ghana, knust.edu.gh

**Keywords:** burnout, emotional exhaustion, healthcare professionals, incident reporting, nurse managers, nurses, patient safety, toxic leadership behavior

## Abstract

**Introduction:**

Patient safety is a vital component of healthcare delivery, and identifying and reporting incidents are essential to raising the standard of care. Nurse managers are noted in some organizations to display a toxic leadership style, which exacerbates nurses’ already existing emotional exhaustion. Nurses’ emotional exhaustion may harm the reporting of patient safety incidents. Using the job demands–resources theory as the primary guiding framework, the study assessed the relationship between emotional exhaustion and patient safety incident reporting among nurses while examining the mediating role of toxic leadership of nurse managers in a teaching hospital in Ghana.

**Methods:**

The study employed a cross‐sectional design. Using convenience sampling, 282 nurses from the various units of a teaching hospital completed the Maslach Burnout Inventory, the Toxic Leadership Behavior of Nurse Managers Scale, and the Survey on Patient Safety Culture tools. Pearson moment‐product correlation and Hayes’ PROCESS Macro mediation analyses were conducted to examine the effects of toxic leadership behavior of nurse managers on the relationship between nurses’ emotional exhaustion and patient safety incident reporting. Analysis was performed at a significance of 0.05.

**Results:**

Close to half (43.6%, *n* = 123) of participants indicated their likelihood of reporting patient safety incidents in their units. There was a significant negative correlation between patient safety incident reporting and emotional exhaustion (*r* = −0.617, *p* < 0.01). The results also showed a significant indirect effect of emotional exhaustion on patient safety incident reporting through nurse managers’ toxic leadership behavior (*c* = −0.0590, 95% CI: [−0.0818, −0.0370]).

**Implications for Nursing Management:**

The toxic leadership behavior of nurse managers acts as a mediator, exacerbating the negative relationship between emotional exhaustion and patient safety incident reporting. The results highlight the need to address emotional exhaustion and toxic leadership behavior of nurse managers to foster a culture of transparent patient safety incident reporting. Improving reporting and minimizing the harmful effects of emotional exhaustion require addressing the role of toxic leadership.

## 1. Introduction

Patient safety incident (PSI) reporting plays a critical role in healthcare organizations by facilitating the identification, analysis, and prevention of adverse events [1]. Nurses, as frontline care providers, are essential to the reporting process because they often witness and respond to incidents directly [2, 3]. A significant challenge of PSI is underreporting by healthcare staff in healthcare settings. Numerous studies have shed light on the extent of underreporting and its implications [4, 5]. For instance, according to Dirik et al. [6], 81.8% of PSIs are identified during care delivery, but only 49.4% of these incidents are reported. These statistics indicate a substantial gap between the number of incidents identified and reported, suggesting a significant underreporting problem among nurses.

The underreporting of PSIs by nurses has far‐reaching implications for patient outcomes. According to Binkheder et al. [7], there is a strong correlation between underreporting and PSI: incidents that are not reported are more likely to result in patient harm than those that are reported. Similarly, underreported incidents led to delayed interventions, repeated errors, and increased patient morbidity and mortality [8, 9]. These findings emphasize the urgent need to address underreporting to safeguard patient safety.

Several barriers contribute to nurses’ challenges with PSI. Mascarenhas et al. [10] and Vrbnjak et al. [11] indicate that fear of blame and reprisals, lack of time, and uncertainty about what constitutes reportable incidents were among the primary barriers to PSI reporting. In addition, organizational culture, lack of trust in the reporting system, and workload pressures are significant impediments to reporting. These listed factors have been shown to contribute to emotional exhaustion (EE), with significant implications for patient safety [12–14].

The demanding nature of the nursing profession, characterized by heavy workload, time pressures, and frequent exposure to emotionally charged situations, puts nurses at a higher risk of burnout [15, 16]. EE, one of the dimensions of burnout, refers to a state of chronic fatigue and depletion of emotional resources, which can significantly impact nurses’ well‐being, job performance, and patient safety outcomes [17]. Studies consistently demonstrate that EE is highly prevalent among nurses. According to Chemali et al. [18], EE is estimated to range from 40% to 60% across diverse healthcare systems and cultural contexts. In Africa, although research on EE among African nurses is limited, Dubale et al. [19] found that 45.8% of nurses reported EE symptoms. It is, however, essential to note that prevalence may vary across African countries due to differences in healthcare systems, resources, and cultural factors. Similar trends have been reported in Ghana; therefore, this high prevalence underscores the need for targeted interventions to address EE among nurses [20, 21].

Various factors, including workload, organizational climate, interpersonal conflicts, role ambiguity, and inadequate social support, influence nurses’ EE. In addition, long working hours, high patient‐to‐nurse ratios, and insufficient staffing are everyday stressors for nurses worldwide [22, 23]. Similarly, factors such as low salaries, poor working conditions, and inadequate access to support systems [24], exposure to patient suffering [25], ethical dilemmas [26], and the emotional intensity of providing care [27] exacerbate EE among nurses.

Significantly, emotionally exhausted nurses may struggle to provide empathetic and compassionate care, thereby compromising patient safety outcomes [28]. Underreporting PSI due to EE can result in missed opportunities for organizational learning, quality improvement, and prevention of future adverse incidents [29]. A culture of silence and fear can prevail, hindering effective communication and collaboration among healthcare professionals, thereby compromising patient safety [30, 31].

The link between EE and PSI reporting has been a growing area of interest in nursing research. Nurses experiencing EE may face significant barriers in engaging in PSI reporting due to reduced motivation, impaired cognitive functioning, and diminished attention to detail [19]. Moreover, EE can lead to a decreased sense of personal accomplishment, which may further undermine nurses’ willingness to report PSI, as they perceive it as an additional burden [32, 33].

While the influence of EE on PSI reporting is well documented, little attention has been given to the potential factors that may exacerbate or ameliorate this relationship. One potentially significant mediator is the toxic leadership behavior of nurse managers (ToxBH‐NM).

In recent years, toxic leadership has become increasingly prevalent in nursing and other healthcare professions. It encompasses actions such as abusive supervision, lack of support, and unfair treatment. According to Labrague [34], toxic leadership is a negative form of leadership because the leader consistently engages in damaging behaviors that directly or indirectly affect individuals or the organization. Other studies, in addition, assert that toxic leaders employ destructive and dysfunctional actions or behaviors, including humiliating, intolerant, self‐promoting, and narcissistic behaviors, to promote a hostile work environment in the organization, exacerbating EE among nurses [35, 36].

Again, findings from Ofei et al. [36] indicate that the presence of toxic leaders may hinder nurses’ emotional well‐being and job satisfaction, further eroding their willingness to report PSI. Nurses who perceive their leaders as unsupportive, disrespectful, or dismissive may fear retaliation or face barriers to open communication, thereby impeding their reporting behavior. Consequently, the negative impact of EE on PSI reporting may be amplified in the presence of TLB, ultimately compromising patient safety outcomes [34].

There has been limited research on the potential mediating role of TLB among nurse managers in the relationship between EE and PSI reporting among nurses. In many African health systems, including Ghana, PSI reporting is hindered by hierarchical workplace cultures. Toxic leadership, characterized by intimidation, authoritarianism, and poor support, may intensify nurses’ EE and further suppress open reporting, yet this dynamic remains unexamined, mainly in the region. By focusing on Ghanaian nurses and positioning TLB as a mediator between EE and PSI reporting, the study offers a contextually grounded and original contribution to understanding how leadership behavior and workforce well‐being shape patient safety practices in resource‐constrained settings. The study aimed to assess the influence of TLB of nurse managers on the association between EE and PSI reporting among nurses. The findings of this study will contribute to a deeper understanding of the interactions among EE, toxic leadership, and PSI reporting in nursing practice and across the healthcare industry.

### 1.1. Theoretical Framework

The study was guided by the job demands–resources (JD‐R) theory as the primary lens. The JD‐R conceptualizes the work environment as comprising demands that deplete energy and resources and as buffering strain [37]. Toxic leader behavior increases cognitive‐emotional load (fear, moral distress, etc.) and reduces access to resources (support, autonomy, etc.). Toxic leadership is theorized as a social/organizational demand (and hindrance stressor) that erodes psychological resources and increases strains, leading to EE. In turn, EE reduces nurses’ willingness and capacity to engage in discretionary safety behaviors, such as PSI reporting, which require time, attention, and a willingness to incur interpersonal risk.

To reinforce how resource loss, i.e., EE, may affect PSI reporting, the study drew from the Conservation of Resources theory [38]. When resources are depleted, nurses are more likely to withhold reporting to conserve remaining psychological resources. In addition, perspectives from Voice and Silence theory [39] reinforce the argument that under toxic leadership, the perceived cost of reporting outweighs potential benefits, amplifying silence. Nursing units with higher levels of toxic leadership will show greater EE and lower PSI reporting, with weaker effects in contexts with strong contextual resources [40]. Hypotheses 1 and 2 correspond to JD‐R’s resource depletion mechanism, while Hypothesis 3 corresponds to the Conservation of Resources theory. Hypothesis 4 corresponds with the augmentation of the JD‐R and Voice and Silence theories.

This theoretical positioning provides a multilevel lens where leadership operates at the unit/organizational level, while EE and PSI reporting decisions sit at the individual level. By examining how the TLB of nurse managers influences PSI reporting through EE, this framework yields actionable levers for organizational interventions that either exacerbate or buffer these effects.

### 1.2. Hypotheses

 Hypothesis 1: EE has a positive association with TLB.
 Hypothesis 2: TLB is negatively associated with PSI reporting.
 Hypothesis 3: EE has a negative association with PSI reporting.
 Hypothesis 4: TLB of nurse managers mediates the relationship between EE and PSI reporting.

## 2. Methods and Materials

### 2.1. Study Design

The study employed a cross‐sectional design from March to May 2023. Eight directorates in the Komfo Anokye Teaching Hospital (KATH) were selected as study sites. The hospital is the second‐largest in Ghana and employs 2289 nurses and midwives. It has a capacity of 1000 beds, a complex management hierarchy, and serves as a teaching hospital in Ghana. The study was reported in accordance with STROBE reporting guidelines.

### 2.2. Population

The population studied was the nursing workforce, comprising individuals at different ranks and qualifications who worked across various directorates at KATH. The inclusion criteria for this study were nurses and midwives with at least a year of clinical experience and who were available at the clinical site during the study.

### 2.3. Sampling Technique and Sample Size

Convenience sampling was used to recruit nurses who met the study’s inclusion criteria. This sampling strategy ensured that readily available nurses participated in the survey. A power analysis using the G∗power (V3.1) for a priori sample size estimation for the regression step testing the incremental variance explained by the mediator (linear multiple regression: fixed model, *R* ^2^ increase) was used to estimate the sample size [41]. Based on prior work and aiming to detect a small effect size (*f*2 = 0.10), with alpha set at 0.05 and five predictors, G‐power indicated that a sample of 204 nurses would be sufficient to achieve 95% power. The small estimated effect size was chosen to ensure that a large sample is collected to detect a meaningful correlation between variables.

### 2.4. Measures

Three self‐report scales, including the Maslach Burnout Inventory (MBI), the ToxBH‐NM Scale, and the Survey on Patient Safety Culture (SOPS), were adopted for this study. Sociodemographic data, including unit, age, gender, rank, hours per week, years at the hospital, and years at the current unit, were also collected.

#### 2.4.1. EE

EE among nurses was measured using the MBI questionnaire [42]. The 7‐item EE dimension, rated on a Likert scale from 0 (*never*) to 6 *(everyday*), was used. Higher scores (> 17.68) indicate higher levels of EE. The Cronbach’s alpha coefficient as used in other studies ranged between 0.83 and 0.94 [43, 44].

#### 2.4.2. ToxBH‐NM

The nurses’ perception of toxic leadership of their nurse managers was evaluated using the ToxBH‐NM scale [45], which was composed of 30 items categorized into four domains, including intemperate (*n* = 15 items), narcissistic (*n* = 9 items), self‐promoting (*n* = 3 items), and humiliating (*n* = 3 items) behaviors. Each item was rated on a five‐point Likert scale (1 = not at all to 5 = frequently). The composite mean score for ToxBH‐NM can be characterized as nontoxic (1.0–2.2), moderately toxic (2.3–3.6), or severely toxic (3.7–5.0). The Cronbach alpha for ToxBH‐NM, as reported in other studies, ranged from 0.75 to 0.96 [34, 35].

#### 2.4.3. PSI Reporting

The reporting PSI was measured using the SOPS Hospital Survey 2.0 questionnaire [46]. The instrument had two items rated on a 5‐point Likert response scale of 1 (*never*) to 5 (*always*). *“When a mistake is caught and corrected before reaching the patient, how often is this reported?” and “When a mistake reaches the patient and could have harmed the patient, but did not, how often is this reported?”* The scale’s Cronbach’s alpha after pretesting was 0.91.

### 2.5. Data Collection

The survey was conducted from March to May 2023. Before the study, participants were informed of the aim and provided written informed consent. Every part of the study was explained thoroughly to all participants. Only participants who met the inclusion criteria were approached directly by the researchers. The paper‐based questionnaires were distributed to participants who consented, and completed, anonymous questionnaires were returned to the researchers on the same day.

### 2.6. Data Analysis

The collected data were analyzed using the Statistical Package for the Social Sciences (SPSS) Version 27. Descriptive statistics were used to characterize the participants. Because nurses were nested within nursing units, intraclass correlation coefficients (ICCs) were first computed to assess the extent of nonindependence. Clustering was trivial, as the ICCs were small (< 0.05). Pearson moment‐product correlation analysis was used to determine the relationships among TLB, EE, and PSI reporting. The PROCESS macro, based on ordinary least‐squares regression, was also used to assess the effects of TLB on the relationship between EE and PSI among nurses. The sociodemographic variables (e.g., age, rank, years at work, and hours per day) were controlled to prevent confounding of the relationship. Model 4 of the PROCESS macro was used to test the mediation model’s significance. The bias‐corrected bootstrap method was used since it has more power than the percentile bootstrap method. In this analysis, 5000 bootstrap samples were used; if zero did not appear in the bias‐corrected bootstrap, the point estimate was deemed significant at the 95% confidence level [47, 48]. All study variables were tested for multicollinearity, and the analysis was conducted at a 0.05 significance level.

### 2.7. Ethical Consideration

According to the Helsinki declaration, ethical approval was obtained from the Committee on Human Research, Publication, and Ethics (CHRPE/AP/359/23) of the Kwame Nkrumah University of Science and Technology, Kumasi, Ghana. All identifying details of participants were presented as those of anonymous individuals to maintain confidentiality. All participants were allowed to participate voluntarily with a detailed explanation of the study. All participants were asked to sign a written consent form and, if possible, confirm verbally. Participants were informed that they could opt out at any time during the study.

## 3. Results

### 3.1. Sociodemographic Characteristics of Participants

This study examined the demographic characteristics of nurses, including their unit, age, gender, rank, years of hospital employment, and years of employment in the current unit. A total of 282 nurses participated in the study with a response rate of 75.13%. The mean age of the participants was 33.24 (±5.46). The majority of participants (83.7%, 236) were females. The majority of participants were senior nursing/midwifery officers (*n* = 70, 25%) and worked in the emergency unit (*n* = 78, 27.8%). A bulk of the participants had 4 years of work experience, which constituted 40 participants (14.7%). While 50.2% had worked for 1–5 years, more than half (57.1%) worked 30–40 h a week. Table 1 details the sociodemographic characteristics of participants.

**TABLE 1:** Sociodemographic characteristics of nurses.

Variable	Category	*N*	%	*M*	SD
Age				33.24	5.47

Gender	Male	46	16.3		
	Female	236	83.7		

Rank	Enrolled nurse	17	6.0		
	Staff nurse/midwife	47	16.6		
	Senior staff nurse/midwife	60	21.4		
	Nursing/midwifery officer	59	21.0		
	Senior nursing/midwifery officer	70	25.0		
	Principal nursing/midwifery officer	28	10.0		

Unit	Child Health	13	4.6		
	DDENT	11	3.9		
	Emergency Medicine	78	27.8		
	Family Medicine	31	11.0		
	Internal Medicine	42	14.9		
	Obstetrics and Gynecology	17	6.0		
	Oncology	20	7.1		
	Surgery, Trauma, and Orthopedics	69	24.6		

Years at work	1–5 years	141	50.2		
	6–10 years	62	22.1		
	11 years and above	78	27.7		

Hours per week	Less than 30 h	10	3.5		
	30–40 h	161	57.1		
	More than 40 h	111	39.4		

### 3.2. The Perceived ToxBH‐NM, EE, and PSI Reporting Among Nurses

Table 2 details the EE, toxic leadership of nurse managers, and PSI reporting among nurses. Participants exhibited moderate EE. The composite mean score for toxic leadership was 2.84 (SD = 0.80), indicating a perceived moderate level of toxic leadership among nurse managers. The composite mean scores for the subscales, equally depicted as moderate toxic leadership, were as follows: intemperate behavior (*n* = 3.06, SD = 0.95), narcissistic behavior (*n* = 2.70, SD = 0.86), self‐promoting behavior (*n* = 2.76, SD = 0.69), and humiliating behavior (*n* = 2.62, SD = 0.65). The nurses who participated in the study reported EE (mean:23.79; SD: 14.62). Regarding the rate of PSI reporting, 43.6% (*n* = 123) of participants reported sometimes reporting PSI in their units.

**TABLE 2:** The perceived toxic leadership, emotional exhaustion, and PSI reporting among nurses.

**Scale**	**Subscales**	**Mean**	**SD**

ToxBH‐NM		2.84	0.80
	Intemperate ToxBH‐NM	3.06	0.95
	Narcissistic ToxBH‐NM	2.70	0.86
	Self‐promoting ToxBH‐NM	2.76	0.69
	Humiliating ToxBH‐NM	2.62	0.65
Emotional exhaustion		23.79	14.62

	**Responses**	**Frequency**	**Per cent**

PSI reporting	Most of the time/always	88	31.2%
	Sometimes	123	43.6%
	Never/rarely	71	25.2%

### 3.3. Relationship Between Toxic Leadership, EE, and PSI Reporting

Table 3 details the significant negative correlations between PSI reporting and EE (*r* = −0.617) and TLB (*r* = −0.643). Similarly, there was a statistically significant negative correlation between PSI reporting and intemperate toxic leadership (*r* = −0.668), narcissistic toxic leadership (*r* = −0.525), self‐promoting toxic leadership (*r* = −0.442), and humiliating toxic leadership (*r* = −0.519). However, there was a statistically significant positive correlation between EE and toxic leadership (*r* = 0.794). All correlations were statistically significant at *p* < 0.01. These findings indicate that as perceptions of TLB increase, both EE and underreporting of PSIs also increase.

**TABLE 3:** The relationship between toxic leadership, emotional exhaustion, and patient safety incident reporting.

Variables	1	2	3	4	5	6	7
1. PSI reporting	1						
2. Emotional exhaustion	−0.617^∗∗^	1					
3. Intemperate toxic leadership	−0.668^∗∗^	0.844^∗∗^	1				
4. Narcissistic toxic leadership	−0.525^∗∗^	0.643^∗∗^	0.752^∗∗^	1			
5. Self‐promoting toxic leadership	−0.442^∗∗^	0.531^∗∗^	0.674^∗∗^	0.902^∗∗^	1		
6. Humiliating toxic leadership	−0.519^∗∗^	0.647^∗∗^	0.779^∗∗^	0.856^∗∗^	0.810^∗∗^	1	
7. Toxic leadership	−0.643^∗∗^	0.794^∗∗^	0.946^∗∗^	0.919^∗∗^	0.849^∗∗^	0.895^∗∗^	1

^∗∗^Correlation is significant at the 0.01 level (2‐tailed).

### 3.4. Effects of EE on PSI Reporting as Mediated by TLB of Nurse Managers

**FIGURE 1:**
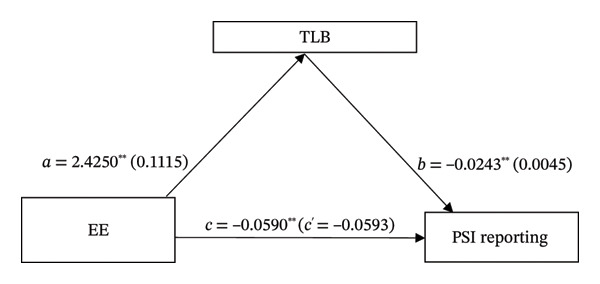
Mediation analysis. The mediating role of TLB in explaining the relation between EE and PSI reporting among nurses (Path 1 in Table 4). *N* = 282; controlled for age and weekly working hours; EE, emotional exhaustion; TLB, toxic leadership behavior; PSI, patient safety incident; *a* = direct effect of EE on mediators; *b* = direct effect of mediator on PSI reporting; *c* = total effect of EE on PSI reporting; *c* ^′^ = direct effect of EE on PSI reporting.

Based on the findings from the correlation study, a mediation analysis was conducted on the effects of EE on the relationship between TLB of nurse managers and PSI reporting. Results as detailed in Table 4 and Figure 1 are summarized as follows: EE has a direct effect on PSI reporting (*a* = 2.2450, 95% CI: [2.2054, 2.6446]), the direct effect of TLB on PSI (*b* = −0.243, 95% CI: [‐0.0333, −0.0154]), and EE on PSI reporting (*c*’ = − 0.0593, 95% CI: [−0.0866, − 0.0320]). These findings support Hypotheses 1, 2, and 3. The results also showed a significant indirect effect of EE on PSI, reported through the TLB of nurse managers (*c* = −0.0590, 95% CI: [‐0.0818, −0.0370]), confirming Hypothesis 4. Overall, the mediation model confirmed that TLB partially mediated the relationship between EE and PSI reporting among nurses.

**TABLE 4:** Results of mediation analyses.

**Paths**	**C**	**SE**	**T**	**95% CI**	**R** ^2^

EE⟶TLB	2.4250^∗∗^	0.1115	21.7409	(2.2054, 2.6446)	0.6305
TLB⟶PSI reporting	−0.0243^∗∗^	0.0045	−5.3636	(‐0.0333, −0.0154)	0.4503
EE⟶PSI reporting	−0.0593^∗∗^	0.0139	−4.2804	(‐0.0866, −0.0320)	

	**Effect**	**Boot SE**	**Boot LLCI**	**Boot ULCI**	

EE⟶TLB⟶PSI reporting	−0.0590^∗^	0.0115	−0.0818	−0.0370	

*Note: N* = 282. The paths were controlled for age and weekly working hours; the displayed effects are standardized.

Abbreviations: EE, emotional exhaustion; PSI, patient safety incident; TLB, toxic leadership behavior.

^∗^Statistical significance level of *p* < 0.05.

^∗∗^Statistical significance level of *p* < 0.01, the indirect effect is significant (^∗^) when the 95% CI does not include 0; SE, bootstrap regression standard error; *R* ^2^.

## 4. Discussion

Nursing, as a demanding profession, often involves high levels of stress and intense patient interactions. Consequently, nurses are at risk of EE. The study examined the mediating role of toxic leadership of nurse managers on the relationship between EE and PSI reporting.

Several studies have investigated the prevalence of EE among nurses, with most nurses having experienced high levels of EE in Italy [49], Iran [50], Jordan [51], Switzerland [52], and the United States [53], indicating it as a significant issue within the profession. The prevalence is similar to that of this study and highlights the widespread nature of EE among nurses. This increased prevalence suggests that EE among nurses may be on the rise, possibly due to escalating work demands, staffing shortages, and other organizational factors [20, 21]. Again, many studies reiterate that EE can lead to decreased motivation, increased cynicism, and reduced engagement [54], which may contribute to nurses’ reluctance to report PSIs. The increase in prevalence rates in recent studies suggests the need for continued attention and interventions to address this problem.

Moreover, the toxic leadership among nurse managers in the current study is consistent with previous research, which found that nurses reported experiencing toxicity from their managers [36, 55, 56]. This finding aligns with that of Reyhanoglu and Akin [57], suggesting a persistent issue in healthcare organizations. The findings of this study reinforce the significance of addressing toxic leadership among nurse managers. Though Labrague [34] asserts that the prevalence of toxic leadership has remained relatively moderate over time in most high‐resource countries, there is a need for continued efforts to mitigate its impact on nurses and healthcare organizations. The findings highlight that the TLB of nurse managers, such as abusive supervision, lack of support, and arbitrary decision‐making, exacerbates the reluctance to report PSI [58]. Organizations should prioritize the development of supportive leadership programs and training initiatives to ensure positive leadership behaviors among nurse managers. By implementing evidence‐based interventions, healthcare organizations can create a healthier work environment and promote nurses’ well‐being.

The study projected that most nurses are reluctant to report PSIs. Similar findings from Hewitt et al. [59] and Yusuf and Irwan [60] found that nurses often hesitate to report incidents due to fear of blame or punishment. This fear is rooted in organizational cultures that prioritize individual accountability over systemic improvement [61]. In addition, Amaniyan et al. [62] highlighted nurses’ lack of awareness of PSI reporting’s importance, leading to underreporting. These findings demonstrate possible fear of repercussions and inadequate education, both of which serve as significant barriers to effective PSI reporting [63].

Furthermore, the study’s findings are consistent with those of Godwin [64], who found that organizational support and leadership play a vital role in promoting PSI reporting. When nurses perceive a supportive culture and feel that their reports are taken seriously, they are more likely to report incidents. Consistent with Falcone et al. [61], this study found that nurses’ reluctance to report PSIs may stem from a punitive reporting culture and inadequate managerial support, both of which are reinforced under toxic leadership.

Regarding the impact of nurse manager toxic leadership on the relationship between EE and PSI reporting, the study found that toxic leadership can lead to adverse outcomes for nurses. This position aligns with the findings of Sun et al. [65] and Zhang et al. [66], who reiterated that abusive supervision, which can be considered a form of toxic leadership, positively relates to EE. Labrague, Nwafor, et al. [45], and Boamah [67] found that transformational leadership, characterized by supportive and empowering behavior, was associated with higher levels of PSI reporting. In contrast, toxic leadership in the form of intemperate, narcissistic, self‐promoting, and humiliating is associated with lower levels of PSI reporting [34]. This study suggests that toxic leadership may hinder the reporting of PSI among nurses. These findings highlight that, even in a resource‐constrained environment such as Ghana’s tertiary hospitals, the psychosocial effects of leadership and workload mirror those observed internationally.

The mediation analysis confirmed that toxic leadership significantly explained part of the relationship between EE and reduced PSI reporting (*β* = −0.0243, *p* < 0.01). This finding is consistent with Ferreira et al. [68] and Opoku et al. [69], who reiterate that toxic leadership by nurse managers contributes to EE and may impair nurses’ ability to report PSI. A similar finding was also reported in the current study, which examined the mediating role of toxic leadership in the relationship between EE and PSI reporting among nurses. This position suggests that toxic leadership by nurse managers can contribute to EE among nurses, which may, in turn, affect their ability to report PSI.

## 5. Implications for Nursing Management

The findings from this study have practical implications for healthcare organizations and nurse managers. This study established that toxic leadership mediates the relationship between EE and PSI reporting among nurses. These dynamics inhibit transparency, reinforce fear‐based cultures, and threaten patient safety. Recognizing the detrimental effects of EE and the toxic leadership of nurse managers on PSI reporting, organizations should prioritize efforts to mitigate EE and foster a positive work environment. Addressing EE requires a multifaceted approach that encompasses individual, organizational, and systemic interventions. Individual‐level interventions include stress management techniques, self‐care strategies, and mindfulness‐based interventions. Organizational‐level interventions involve fostering supportive work environments, improving nurse–patient ratios, and providing opportunities for professional development and growth. Systemic interventions encompass policy changes and healthcare reforms that recognize and prioritize nurses’ well‐being as essential to delivering high‐quality patient care. Moreover, nurse managers should be trained to display supportive and transformational leadership behaviors, fostering an environment of trust, open communication, and psychological safety. Encouraging reporting and addressing concerns about retaliation are critical steps toward promoting patient safety and quality care.

In addition, this study highlighted the prevalence of perceived toxic leadership of nurse managers and its detrimental effects on nurses’ well‐being. This finding demonstrates the study’s practical significance by suggesting that leadership style is not merely a contextual factor but a critical mechanism shaping nurses’ reporting behavior. Healthcare organizations must prioritize cultivating positive leadership behaviors and creating a supportive work environment that promotes nurse satisfaction and retention. Addressing toxic leadership among nurse managers is essential to ensuring high‐quality care and the overall success of healthcare organizations.

## 6. Limitations

Self‐report measures, which may introduce response bias and social desirability, were used in the research. However, the validated tools were improved to ensure the reliability and validity of responses. The association between EE, toxic leadership among nurse managers, and PSI reporting may be better understood through longitudinal designs and by exploring other factors, such as work satisfaction and organizational climate. Future research should therefore examine interventions that address EE and leadership development to improve nurses’ PSI reporting practices.

## 7. Conclusion

Toxic leadership of nurse managers inhibits PSI reporting, stifles a culture of openness, and promotes a blame‐and‐fear mentality. The reporting culture is further deteriorated by the fact that nurses who are emotionally exhausted are more likely to hold back on reporting PSI, because they are worried about negative consequences. This lack of PSI reporting makes it difficult to identify and address safety hazards that endanger patient health. Healthcare organizations can promote a culture of safety, transparency, and continuous learning by addressing the toxic leadership of nurse managers and EE, thereby enhancing patient outcomes and the quality of healthcare delivery.

## Author Contributions

Collins Atta Poku, Yvonne Akpene Ahiadzese, Hadassah Korkor Oyortey, Sarah Naa Odarkai Lamptey, and Jeremiah Asante conceptualized and designed the study method. Collins Atta Poku, Yvonne Akpene Ahiadzese, C.O.A., Hadassah Korkor Oyortey, Sarah Naa Odarkai Lamptey, and Jeremiah Asante collected, analyzed, and interpreted the data. Collins Atta Poku, Daisy Afrah Lumor, and Doris Hagan drafted the original manuscript.

## Funding

This work was not supported by any funding agency.

## Disclosure

All authors read, revised, and approved the final manuscript for submission.

## Conflicts of Interest

The authors declare no conflicts of interest.

## Data Availability

The data that support the findings of this study are available from the corresponding author upon reasonable request.
